# Lacerated Tongue Injury in Children

**DOI:** 10.5005/jp-journals-10005-1007

**Published:** 2008-12-26

**Authors:** Usha Mohan Das, Prahlad Gadicherla

**Affiliations:** 1 President, Indian Society of Pedodontics and Preventive Dentistry (ISPPD), Principal, Prof. and Head, Dept. of Pedodontics and Preventive Dentistry, KR Road VV Puram, Bangalore, Karnataka, India; 2 Former Postgraduate Student, Department of Pedodontics and Preventive Dentistry, VS Dental College and Hospital, KR Road VV Puram, Bangalore, Karnataka, India

**Keywords:** Tongue biting, Tongue laceration.

## Abstract

Other than in patients suffering from epilepsy, tongue lacerations are rare. Most commonly, these injuries occur when the tongue is between the teeth and a fall or blow occurs. They cause parents to panic and the child to cry uncontrollably with blood, tooth and soft tissue debris in the mouth. The presenting characteristics of the patient and injury as well as the treatment rendered and its outcomes are described.

## INTRODUCTION

Tongue biting in seizures has been extensively described. Lateral tongue biting is 100% specific for epileptic seizures and may be helpful in ifferentiating seizures from syncope and pseudoseizure.^1^

The most common location for a lacerated tongue injury is the anterior dorsum of the tongue. A fall at home is the most common situation. The next most common locations are the mid dorsum and anterior ventrum. The frequency of injury reduces from anterior to posterior on both surfaces.^2^

Hemorrhage and disfigurement are the two most common concerns in these injuries, although loss of function, infection,and swelling that compromises the airway are also mentioned as sequelae.

The purpose of this article is to provide an insight on lacerated tongue injury in pediatric patient and to assist the clinician in the management of this unique and highly specialized area of traumatology.

The largely anecdotal literature on tongue lacerations can confuse clinicians. Andreasen and Andreasen^3^ suggest suturing both dorsum and lateral border injuries. Powers et al^4^ suggest loosely suturing tongue wounds and placing deep sutures in layers. Donat et al^5^ recommend suturing only wounds larger than 2 cm or when hemorrhage is a concern. English 6 agrees that small lacerations need not be sutured when wound margins are in good approximation. Touloukian^7^ warns that suturing may predispose the tongue to invasive, closed space infection.

## CASE REPORT

**Fig. 1:**
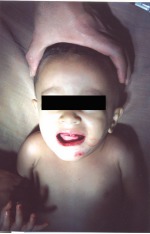
Preoperative

A 1½ year patient presented to the department with a fall from the first floor of the building while playing (Fig. 1).

The patient was conscious, but irritable. There was no history of convulsions, vomiting, loss of consciousness, seizures, neck stiffness or injury to any other part of the body.

### General Examination

- Pulse 78/min
- Bleeding from mouth (soft tissues).

### Intraoral Examination

**Teeth present:** 51,61 

71,81 (Fig. 2)

Laceration on left lateral border of tongue measuring

1½ cm × 1 cm × 0.5 cm.

**Treatment done:** Tongue suturing done with catgut 3-0 under monitored anesthetic care (MAC) (Fig. 3).

- Patient premedicated with Inj midazolam 0.5 mg
- Inj pentazocine 5 mg Inj metaclopropamide 2.5 mg
- Postoperative Instructions: Liquid diet
- Maintain proper oral hygiene (chlohex mouth wash)
- Mox syrup tid × 5 days
- Crocin Syrup 1 tsp SOS

**Follow-up:** Healing Satisfactory Figs 4 to 6.

**Fig. 2:**
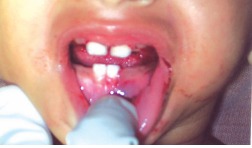
Teeth present : 51, 61, 71, 81

**Fig. 3:**
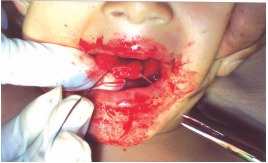
Intraoperative suturing

**Fig. 4:**
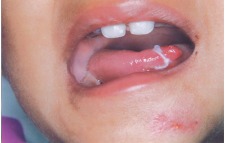
Follow-up 3 days catgut in place

**Fig. 5:**
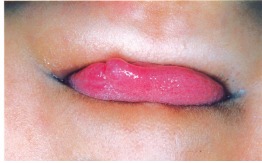
Healing satisfactory (after)

**Fig. 6:**
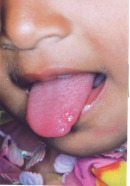
Postoperative (1 Month after)

## DISCUSSION

Management principles for soft tissue injuries are much the same except that treatment should be initiated within hours because healing occurs sooner. Although immature collagen in the childs soft tissue provides very cosmetic results vast majority of time hypertrophic scars and keloids may form in this patient population.^8^

A wound located on the dorsal surface of the tongue should always be examined for a ventral counterpart. If there are concomitant crown fractures, pieces of teeth or restorations fragments may be located within the wound.These fragments can be revealed by a radiographic examination with an exposure time 25% of normal exposure time.

Treatment principles for penetrating tongue wounds include cleansing of the wound, removal of foreign bodies and suturing of the dorsal and ventral aspects of the lesion. After administration of anesthesia (local, regional or general) foreign bodies are retrieved as, it is very important to prevent infection and/or scar formation the wound cleansed with saline, and the wound sutured tightly. Administer antibiotics if indicated. Suture removal after 4-5 days for lateral border wounds buried resorbable sutures are sometimes indicated in order to approximate the wound edges and relieve tension on the mucosal sutures.

The tongue has a rich blood supply, and injuries to the tongue or the floor of the mouth may cause serious hemorrhage that potentially threatens the airway. the airway may become compromised some time after trauma to the tongue or lacerations of the floor of the mouth if veins are damaged, resulting in swelling of the tongue into the oropharynx.

Deep lacerations should be closed in layers, with chromic catgut sutures in the muscle layers to prevent formation of a hematoma.

Reconstruction of laceration or avulsion tongue injuries is not usually required; primary wound healing often occurs rapidly because of the rich vascular supply of the tongue. only with lacerations larger than 2 cm or with difficulties in obtaining hemostasis is it necessary to effect closure. In the child, this must usually be performed with the use of heavy parenteral sedation or under general anesthesia, because it is difficult to immobilize the tongue for local anesthetic infiltration and subsequent suturing, using absorbable suture. Loss of lateral tongue or tongue tip from extensive injury usually produces no permanent deficit, since the tongue hypertrophies to rebulk itself in a period of 6 months. Injuries involving the tongue base are more likely to affect function in the event of hypoglossal nerve injury or late fibrosis from large transecting lacerations. many times a laceration in which a flap of muscle is elevated may be ignored: this results in some distortion of the tongue, which is of concern to the parents and later to the child.

A special group of injuries to the tongue are those that occur in children with coagulopathies. These tend to heal slowly and although they may heal primarily, the process may take weeks in a child who has hemophilia. Suture repair of relatively small injuries in these cases may expedite healing and is advisable since it is necessary to maintain these children with replacement coagulation factors. Dental fabrication of a smooth splint for the prevention of wound irritation by the maxillary teeth is additionally warranted.

For effective function after the proper repair of the lip and tongue, early motion is advisable. Within several days after removal of the sutures, the patient should be executing stretching exercises. These exercises should be continued until the tissues soften and the period of wound contracture has passed.

## What this Case Report Adds?

Tongue lacerations are common among intraoral soft tissues in children and can occur from falls or as penetrating injuries from sticks or other objects.

## Relevance to Pediatric Dentists

A tongue laceration in a child poses a management dilemma for the clinician to as suture or not, especially in a young child when behavior management is an additional consideration. The dilemma is compounded because sedation or even general anesthesia may be required.
